# An Intra-Subject Approach Based on the Application of HMM to Predict Concentration in Educational Contexts from Nonintrusive Physiological Signals in Real-World Situations

**DOI:** 10.3390/s21051777

**Published:** 2021-03-04

**Authors:** Ana Serrano-Mamolar, Miguel Arevalillo-Herráez, Guillermo Chicote-Huete, Jesus G. Boticario

**Affiliations:** 1 aDeNu Research Group, Artificial Intelligence Department, Universidad Nacional de Educación a Distancia, 28040 Madrid, Spain; jgb@dia.uned.es; 2 Departament d’Informàtica, Universitat de València, 46100 Burjassot, Valencia, Spain; miguel.arevalillo@uv.es (M.A.-H.); guillermo.chicote@uv.es (G.C.-H.)

**Keywords:** affective computing, physiological sensors, nonintrusive, learner modelling, user-centred systems

## Abstract

Previous research has proven the strong influence of emotions on student engagement and motivation. Therefore, emotion recognition is becoming very relevant in educational scenarios, but there is no standard method for predicting students’ affects. However, physiological signals have been widely used in educational contexts. Some physiological signals have shown a high accuracy in detecting emotions because they reflect spontaneous affect-related information, which is fresh and does not require additional control or interpretation. Most proposed works use measuring equipment for which applicability in real-world scenarios is limited because of its high cost and intrusiveness. To tackle this problem, in this work, we analyse the feasibility of developing low-cost and nonintrusive devices to obtain a high detection accuracy from easy-to-capture signals. By using both inter-subject and intra-subject models, we present an experimental study that aims to explore the potential application of Hidden Markov Models (HMM) to predict the concentration state from 4 commonly used physiological signals, namely heart rate, breath rate, skin conductance and skin temperature. We also study the effect of combining these four signals and analyse their potential use in an educational context in terms of intrusiveness, cost and accuracy. The results show that a high accuracy can be achieved with three of the signals when using HMM-based intra-subject models. However, inter-subject models, which are meant to obtain subject-independent approaches for affect detection, fail at the same task.

## 1. Introduction

Affective computing can lead to significant improvements in education [1]. There is strong evidence coming from previous research showing that emotions have an important effect on the student’s engagement and motivation and consequently influence learning outcomes [2,3,4]. Previous studies named engaged concentration as the most prevalent affect in a classroom context [2]. Engaged concentration is a state of engagement with a task such that concentration is intense, attention is focused and involvement is complete [5]. Thus, effective detection of the concentration state over time is a crucial task for adaptive learning systems that aim to take proper action in order to improve student engagement.

However, detecting emotions in an educational context is still a challenge. Many previous works have been published to overcome this problem, but there is still no consensus regarding the methods that best suit recognising a particular emotion. Signals from autonomic nervous system (ANS) responses have been widely used due to the significant correlation of their changes with emotional stimuli [6,7]. Thus, changes in heart rate, blood pressure, temperature, breathing, etc. are used as features to build models in affect recognition. This type of signals has been extensively used in many contexts [8], including education [9]. The main advantages of physiological signals over other sources of information such as visual resources are as follows: (1) the ANS is mostly automatically and involuntarily activated, and thus, since physiological signals are the result of ANS activity, they are fresh information sources and cannot be easily faked; (2) physiological signals are also more effective at extracting individual traits from each subject. However, this type of signal presents several drawbacks: (1) their features are less generalizable across individuals than others such as the activation of facial muscles; (2) they are more difficult to collect and their collection is usually more intrusive for the subject, which can condition the experiments and their results; and (3) the sensor costs can be relatively more expensive than when using other capturing methods, such as vision-based systems. These aspects have been a limitation to the use of this type of signal in both real interactive environments and typical educational settings.

Because of the above limitations, it is crucial to provide cost-effective approaches for detecting each person’s emotional state from signals that offer more information on relevant states in the given context and can be unobtrusively collected. If intrusiveness is reduced, accuracy is maximised and the cost is minimised, the use of physiological signals may become a practical alternative, whether on their own or combined with other detection methods. This justifies the increasing interest in Commercial-Off-The-Shelf (COTS) wearables for physiological monitoring in different contexts [10,11], including education [12]. The key benefit of those devices are their wearability and thus their low intrusiveness. The use of different low-cost wearables that measure daily a subject’s activity is becoming very popular in our society, especially wristbands for exercise tracking. However, commercial devices are not yet completely ready for research purposes [13,14] because of their low sampling rate, low accuracy and the fact that most of them only provide a few signals that are not enough to predict emotions. In this work, we study the feasibility of using four signals that are considered relevant in affect recognition [15] to detect the concentration state, namely heart rate, skin conductance, skin temperature and breath rate. These four signals can be easily captured by sensors which could be all integrated into low-cost wearables in the near future.

Some previous research in physiological-based affect detection attempted to build subject-independent (inter-subject) models [16,17,18,19]. In such works, a common model is built by considering all data as if it were coming from the same subject, without taking into account the particularities of each subject. This approach has the advantage that the model can be used with unseen subjects. However, such a generalisation can have a negative effect on detection accuracy, especially when using physiological patterns, which can vary both from subject to subject and across contexts. Another early research approach was to build individual models adapted to each user, known as subject-dependent (intra-subject) models. The benefits of subject-dependent models have been proven in several settings that use Electroencephalography (EEG) signals [20,21,22] or keyboard and mouse signals [23], to name a few. This approach offers better detection accuracy by building models that are specifically tailored for each subject. However, their construction requires a high amount of training data for each individual, and the resulting model cannot be used for individuals other than the one they were targeted to.

We aim to advance methodological issues and to open the way towards the development of new commercial devices that can be integrated in affect-aware user-centred adaptive systems in real-world educational scenarios. With this in mind, we study the potential use of intra-subjects and inter-subject modelling approaches when they are combined with multimodality fusion methods and fed with a common set of physiological signals that are relatively easy to capture by using nonintrusive low-cost devices. We focus on the concentration state of the subject, which has a particular interest from an educational perspective. Thus, we use Hidden Markov Models (HMM) in order to capture the dynamic nature of a cognitive state that builds up along the time axis. HMMs have been widely used in speech synthesis and lately in facial expression synthesis and have been also recently proposed to be used for affect recognition when using physiological signals [24,25] because of their ability to model nonstationary signals or events [25]. The results of our analysis suggest that a reasonable accuracy can only be obtained when intra-subject models are used. Furthermore, they outline the need to intensify research efforts on techniques that enable the automatic construction of intra-subject models.

The rest of this paper is organised as follows. Section 2 describes some previous work that relates to the methods and results presented throughout the paper. Section 3 focuses on the experimental design, also covering the acquisition system, data collection, data labelling and data preprocessing steps. Section 4 reports the experimental results, which consist of evaluating the intra-subject and inter-subject models and comparing the performance of different multimodal fusions of the four physiological signals. Section 5 provides a discussion of the results. Finally, the conclusions and future research activities are drawn in Section 6.

## 2. Related Work

The multiple reviews on the affect recognition field, e.g., [8,26,27,28,29], are a clear indicator of the importance of the topic and the level of research activity that has been reached. Affect recognition has been successfully applied to marketing [30,31], health [32,33], cognitive assistants [34] and more recently learning systems [35,36,37], including methods to automatically recognise student’s engagement [38].

Most of the previous works focused on a less accurate sensor-free approach to restrict intrusiveness and to reduce costs, as in [39,40,41,42,43,44], or were based on visual sources of information, such as facial expressions or eye-tracking, e.g., [18,38,45,46]. Such sources have the advantage that they generalise well between users and enable the construction of inter-subject models. Many other works rely on a multimodal setup. According to D’mello and Kory [47], affect recognition systems based on multimodal data tend to be almost 10% more accurate than their unimodal counterparts. However, sensor-based systems tend to be more intrusive and costly and their applicability is reduced in a real-world educational scenario.

In this review section, we focus on previous works that use physiological data for affect recognition. Feature selection is a crucial step to accurately recognise affective states using physiological signals. Furthermore, the obtrusiveness of the system can also influence detection accuracy. Thus, we have focused not only on works that study student engagement but also on a set of works that use physiological signals to recognise different emotions through different contexts. There are many between-study differences in the literature review in terms of a number of factors that makes it difficult to draw broad conclusions. In our comparison, we have selected the following factors:

- Target emotion. This factor describes the emotion to be detected by the system proposed in the related work. Both categorical and dimensional models are considered in this factor.
- Physiological signals that were used to model the affective state of the user. Different combinations of physiological signals are used across the related works. Some of them are easier to capture and less intrusive than others in a real-world scenario, e.g., some can be obtained from the subjects’ wrist, while others require more intrusive devices, such as a headset or a clamp in the finger.
- Methods/classifiers used.
- Highest accuracy obtained in the study. When the study compares different approaches, such as different signals, modelling approaches or classifiers, the best accuracy reported is included in this review.
- Dependency of the model on the subject. This indicates whether the study follows an intra-subject or inter-subject approach.
- The number of subjects involved in the study. The number of subjects is especially important for inter-subject approaches in order to manage better generalisation results. In contrast, intra-subject approaches simply require a large amount of data from the same individual, and the number of subjects is only relevant to check the consistency of the approach across the many participants.
- Labelling of emotions. This indicates if the labelling is self-reported by the user, determined from the stimulus used to elicit emotions or labelled by an expert.

Table 1 shows a summary of the literature survey of recent works in affect recognition that make use of physiological signals, indicating the aforementioned factors for each case. As in any classification problem, note that the best accuracy reported in each work has to be contextualised according to the number of emotions considered, as the prediction task becomes more challenging as the number of possible states increases.

### 2.1. Emotion Representation

Regarding emotion representation, some works used a dimensional representation whereas others used a categorical one. Within the ones that chose a dimensional representation [48,49,50], most of them adopted the two dimensional model proposed by Russell [51], with valence and arousal as the two main dimensions of affect. On the other side, many works that use a categorical definition of the affective state consider the 6 basic emotions identified by Ekman [52], namely anger, surprise, happiness, disgust, sadness and fear, or a subset of them [53,54,55,56]. However, as stated in [57], these basic emotions are quite infrequent in educational contexts when using learning software tools. Instead, there are other more common feelings that the user experiences during learning tasks, such as boredom, engaged concentration, anxiety, confusion or frustration. Two works [58,59] and the one presented in this paper are focused on non-basic states. These non-basic states only share a few features commonly attributed to basic emotions, and hence, their detection is considered a more challenging problem.

### 2.2. Physiological Signals Selected

Selecting an appropriate set of signals and features that effectively helps discriminate between affective states is a task crucial for affect recognition. Different physiological measures were used in the reviewed works: Galvanic Skin Response (GSR), also named Skin Conductance (SC), ElectroDermal Activity (EDA) or ElectroDermal Response (EDR); heart rate variability (HRV), which can be obtained from electrocardiography (ECG); electroencephalographs (EEG); Skin Temperature (ST); Breath Rate (BR); Electromyogram (EMG); and Blood Oxigen saturation (OXY). Among them, some signals were not easy to capture, at least in a nonintrusive manner, such as EEG, EMG, BVP and OXY. Some researchers centred their works on the use of the features obtained from just one signal to minimise intrusiveness, as in [60]. In that work, the use of off-the-shelf wearable devices that measures EDA to monitor the emotional engagement of students during lectures was studied. Furthermore, the authors investigated the physiological synchrony between teachers and students and how it helps to infer students’ emotional engagement. Another example aiming to provide a feature extraction method for a user-independent emotion recognition system from electroencephalograms (EEGs) is [53]. However, EEG poses strong limitations on the movement of the subjects and, hence, is not applicable in real-world scenarios. The rest of the works used a set of features obtained from more than one physiological signal, with GSR and ECG being the most commonly used in the selected works. Both [58,60] took the intrusiveness of the physiological sensors into consideration in their works. In [58], they concluded the fact that the intrusive physiological sensors used may have influenced their study, affecting the way students engaged with the ITS they used. The system proposed in [60] followed a nonintrusive approach and aimed to continuous gathering sensor data in classroom settings through a wrist-worn and a lightweight device. The rest of the selected works made use of traditional sensors without taking into account their obtrusiveness. Thus, despite the progress made so far in previous works, it is safe to say that there is still a long way to go before affect recognition can be integrated into everyday interfaces and can be more readily deployed into real-world contexts. In the present work, we aim to analyse the use of different nonintrusive sensors towards the integration of an affect detection system in real-world educational scenarios while not influencing student behaviour. Thus, the present study uses a subset of the aforementioned signals, namely, heart rate, breath rate, skin conductance and skin temperature and includes a discussion about the accuracy results obtained with different combinations of them.

### 2.3. Subject Dependency

Some of the selected works focused on building user-independent models for physiological-based affect recognition. This is the case of most [48,50,53,56,59,60] that aimed to build models that have the potential to be generalised to new users as the main advantage. Among these works, the best accuracy was obtained when classifying basic emotions, as in [50], where 90% of accuracy was obtained when discriminating among 5 levels of arousal and valence. In contrast, a lower accuracy was obtained when detecting non-basic emotions, as in [59], where a maximum of 63% of accuracy was obtained when detecting boredom, engagement and anxiety. This lower accuracy was due to the individual differences in affective experience and expression of non-basic emotions. Other works such as [54,55] adopt a user-dependent approach. Two of the selected works, [49,58], compare both approaches. Both of them conclude that better accuracy is obtained with subject-dependent approaches. In [58], they aimed to detect non-basic emotions such as boredom, confusion, curiosity, delight, flow/engagement, surprise and neutral, resulting in engagement, boredom and neutral obtaining the best scores in the user-dependent models [2,5,39]. In [49] the classification of four bidimensional emotions (positive/high arousal, negative/high arousal, negative/low arousal and positive/low arousal) induced by music was studied. It is important to point out that, in this work, they used within-participant cross-validation for the user-independent approach. That means that there were data from the same user in both the training and testing sets. Classification accuracy is expected to decrease from the one reported, CCR = 77%, if a between-subject validation technique is applied, which means that the data from one user appears only in the train or the test set.

### 2.4. Other Aspects

There are other aspects shown in Table 1 that mark some differences between the selected works and that will be summarised in this subsection. Many others could be pointed out, but they are out of the scope of this work, such as the method used for emotion elicitation, the location of the experiment (natural context vs. lab context), and so on.

Another relevant factor to consider in inter-subject approaches is the number of participants in the study, as more participants translate into models with a higher generalisation capacity. In the case of the intra-subject approaches, one way to increase the reliability of the models is to record data from the same user over time and under different situations. The number of subjects involved in the selected work varies from 1 for an intra-subject approach in [54] to 101 for an inter-subject approach in [56].

Regarding classification methods, a wide variety of techniques have been used for classifying affective physiological data. These include k-nearest neighbours, decision trees and SVM, to name a few. According to related work, there is no consensus regarding which one of the algorithms is the most efficient. In this work, we propose the use of HMM, which has been previously used for emotion detection with EEG signals [24,25].

Regarding data annotation, most of the works make use of a self-assessment of the emotion experienced by the subject. Some other works use stimuli to elicit emotions in the subjects, such as music [49,54] or images [50], and the labelling of emotions is objectively determined by the stimulus type. In [58], which used self-reported judgements, they concluded that the interpretation of the labels may vary among individuals and that the results obtained could improve if such self-reported annotations were complemented with judgement from observers or experts. In our study, we used this approach, combining both self-reports and judgements by two experienced experts.

## 3. Experimental Design

### 3.1. Physiological Signals Acquisition

Physiological signals have been widely used to recognise emotions. Some of the signals most commonly used are blood pressure, skin conductance, galvanic skin response, respiration rate and heart rate variability. Some of them are easier to capture than others in a real-world scenario. The nonintrusiveness of the acquisition system is crucial in an educational context when we want to study the student’s behaviour. Some sensors are too intrusive and may disturb correct performance of the learning tasks. For example, those that have to be worn on the fingers prevent the student from using a keyboard or mouse because typing or clicking can provoke signal distortions. Furthermore, the behaviour of the student can be conditioned when using uncomfortable wearables.

In order to avoid the aforementioned problems, in this work, we selected four signals that can be gathered from sensors that minimise this intrusiveness and that could be easily integrated in a low-cost device. Those signals were already identified in [15] as valuable information to identify changes in the subject’s mental state.

The acquisition system, called PhyAs (PHYsiological Acquisition Shield), corresponds to the third iteration of the AICARP (Ambient Intelligence Context-aware Affective Recommender Platform) system, which takes advantage of the environmental intelligence in order to help the learner manage their emotional state while performing learning tasks [62]. In this version, many improvements were introduced, for instance, the accuracy of certain sensors as the skin conductance and the response time of the temperature where revised. Some other improvements aimed to reduce the obtrusiveness of the system, such as the new pulse-oximeter. The selected signals for this study and how they were acquired to minimise the cost and intrusiveness of the system are detailed here:

- **Heart rate**. This signal together with Heart Rate Variability (HRV) and other parameters related to the cardiac circle are measured in many works by an electrocardiogram (ECG) [48,49,50,54,55]. In this work, we used a pulse-oximeter. The pulse sensor that, in previous versions of the PhyAS, used to be a clamp in the finger, as in many studies, was changed by an ear clip sensor, which allows the fingers to be used in writing tests. This sensor can be also placed on the wrist so that it can be easily integrated in a wrist-worn device. However, a better response is obtained from the lobe of the ear. In this version, the acquisition system can provide information regarding instantaneous changes in the heart rate, with similar behaviour to the HRV (Heart Rate Variability) provided by an electrocardiogram, which would be much more intrusive. In this study, we compute the number of beats per minute (BPM).
- **Breath rate**. A relaxation state is generally linked to a decrease in the respiration rate, while momentary cessations of the respiration are linked to tense situations [49]. During respiration, the thorax expands and constricts. Hence, a piezoelectric chest belt is commonly used to measure the respiration pattern [59] of the subjects, as is the case in this study. In particular, we compute the BR as the number of breath cycles taken within a minute (BCPM).
- **Galvanic skin response**. It is very commonly used in affective computing, especially to detect arousal differences. It provides a measure of the resistance of the skin (electrodermal activity or skin conductance), and it is commonly measured at locations with a high density of sweat glands, for example, palm/finger [58], feet [63] or wrist. In some works, this sensor is placed on the fingertips [58], which reduce user mobility to develop writing tasks. In the work proposed in this paper, this sensor is placed on the wrist of the subject to enable user mobility when developing writing tasks.
- **Skin temperature**. Changes in the skin temperature can be measured using an infrared light that does not require contact with the participant’s skin. This is the case of the present study, which uses a sensor placed close to the wrist. Although this sensor is capable of measuring the environmental temperature, we chose not to include such a measure in our analysis.

More details regarding the acquisition system can be found in [15].

### 3.2. Data Collection

A data collection experience was conducted to evaluate the performance of the proposed approach in detecting concentration patterns from the signals. In order to evaluate the applicability of our method in a practical setting, data collection took place in a real school while students interacted with a series of learning tasks. The experience was repeated for a set of 4 different users $U=\{u_{i},1\leq i\leq 4\}$.

The PhyAS was connected to an Arduino Uno to capture the 4 physiological signals at a rate of 10 Hz. At the same time, the student interaction was recorded in video, within a framework that was built to support tracking and labelling from a single-subject experiment, including on-site and offline data labelling. A wider explanation of that framework can be found in [64]. The video and physiological signals recordings were synchronised by using the system’s clock.

The construction of an individual model per subject usually presents a challenge because it is assumed that it requires a large amount of data for each person [20]. However, having the limitations imposed on a real-world learning scenario, where the amount of data from a single user is usually limited [23], we have come up with an affordable setting based on a small number of short-term sessions per user. In particular, to obtain enough information to build intra-subject models, each student participated in 4 sessions. A different learning activity was carried out in each session. The dataset was hence composed of 4 recordings for each of the 4 subjects involved in the experiment. All experiments of this work were carried out following the rules of the Declaration of Helsinki of 1975 [65], and each participant was asked to sign a consent form. This consent form was given to them in advance so that they could read it without any external pressure and contained information about the aim of this research work along with a complete description of the experience. When they signed, they confirmed that they had read the complete form, understood all the information contained therein and authorised us to conduct the experience, including exploitation of the data gathered during the session.

Of the 4 sessions, there were two different types. The first three were focused on detecting the affective state of the user, and the fourth included an additional task where users got feedback when they entered a state of excessive agitation that would hamper their performance. These followed our previous approach [64].

**Figure 1:**
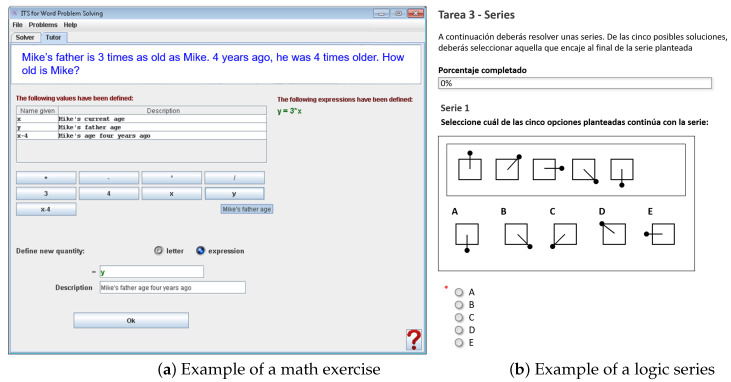
Examples of the exercises proposed to the students during the experiences

In order to guarantee the generalisation of the experience across different applications, exercises related to different learning areas were proposed to the students. Those areas include math, logic and a second language. The first two sessions consisted of a series of math exercises. Those exercises included numerical and algebraic problems, which involved simple arithmetic operations. An example of one of the proposed mathematical problems can be seen in Figure 1a, The third session included a series of logic exercises that the student had to solve. Specifically, they were simple graphical logical series to try to elicit a relief feeling from previous tasks. An example with one of the proposed logical series can be seen in Figure 1b. These first three sessions involved tracking the students with the aforementioned sensors while they performed the requested tasks using a keyboard and a mouse. The fourth session was an oral test in a second language (English). In this scenario, the learners talked for 5 min about a given topic in English as if they were practising for an oral exam. This scenario required competence in public speaking, which is widely used in the literature to induce physiological changes related to different affective states [66,67]. In this fourth session, feedback to the learner was introduced from a band placed on their wrist that vibrated when the user entered a zone of nervous excitement close to stress.

The first three sessions focused on detecting the affective state and followed a well-defined protocol in order to ensure that every session was carried out under the exact same conditions. Such a protocol defines all the steps involved in the session. Thus, the session started by entering the workspace of a web browser with the corresponding user and configuring the activity for the day. After that, the infrastructure for the experiment was launched. Then, the participant was welcomed and informed about the experience. Next, the consent form was signed, and the physiological sensors were placed on the participant and tested to verify that they were correctly collecting data. Once everything was ready, the data collection started and lasted 9 min, which were distributed as follows: 2 min with no activity, 5 min solving exercises and 2 more minutes with no activity. During the 2 min of recording before and after the exercise session, the participant was asked to just relax. The purpose of these recordings without activity was to obtain a baseline for the participants. Having a baseline of the subject helps to model their physiological state when they are not cognitively involved. When the data collection was finished, the sensors were removed from the participant and the recording was stopped. Finally, the data was tagged with the support of the participant.

The fourth session, an oral test in a second language, consisted of a voice baseline and two oral activities of 10 min each with an increasing level of difficulty. The students took them in turn. The session started with a brief presentation explaining the experiment to the participant and confirming their consent to use of the data. The 10 min of each activity were distributed as follows: 2 min with the participant reading a text to record the baseline voice, 1 minute for preparation of the test, 5 min of oral presentation and 2 more min with no activity. Upon each activity, the sensors were removed and the experts labelled the collected data with the participant. Between the first and second activities, the action rule parameters that control the aforementioned band were obtained while the subject completed the General Self-Efficacy Scale (GSE) questionnaire [68] and the performance was configured in the recorder. Moreover, after the second activity, the Personal Data questionnaire and the System Usability Scale (SUS) [69] questionnaires were completed. The SUS questionnaire allows the participant to value the experience environment and was used to evaluate the usability of the infrastructure.

### 3.3. Data Labelling

The video recordings were used to manually label the data. The procedure followed for the labelling was the same as in [70]. Two different experts labelled the data set independently. One expert had a psychology background, and the other had 6 years of experience in the affective computing research field. They identified specific moments in the video where the user seemed to have reached one of a set of specific mental states and used the application described in [64] to mark the concrete instant in time where they believed the peak was. Both experts labelled the complete dataset independently and then discussed any disagreement regarding a particular label at a given time. After the labelling consensus, a validation meeting was held with each participant, correcting the labelling where appropriate.

### 3.4. Data Preparation

Out of the labels provided by the experts, we focused our study on the concentration state. This is because of its relation to engagement and because this label showed the highest reporting agreement between the experts and the subjects.

As with most other mental states, concentration does not occur all of a sudden. We hence can reasonably assume concentration on a time frame of 6 s around the identified peaks. Based on this hypothesis, we took each time mark reported as a concentration peak by the experts and isolated the physiological measurements of the 3 s before and after the peak. With a capture rate of 10 Hz, this yielded a matrix of size 4 × 60 (4 physiological signals × 60 measurements per signal) for each concentration label reported, containing information about how the signals evolved until the concentration peak was reached. We call each of these matrices a concentrated sample.

In order to generate non-concentrated samples, we discarded the first second of the physiological signals and chose 6 s disjoint slots that were at least two minutes apart from any identified concentration peak. We also guaranteed a minimum of 6 s separation space between any two non-concentrated samples. The number of positive and negative samples obtained per user by using this procedure is shown in Table 2.

### 3.5. Description of Experiments

There were two issues to analyse throughout the experimentation: the validity of inter-subject and intra-subject models to predict concentration and relevance of the selected physiological features for concentrated state assessment while studying different combinations. In order to analyse both issues, a diversity of experiments were carried out.

#### 3.5.1. User-Dependent vs. User-Independent Models

In this first set of experiments, we aimed to study the performance of both inter-subject and intra-subject approaches in detecting concentration in an educational scenario by using Hidden Markov Models (HMMs) that are trained with physiological data. To this end, we devised 3 different experiments, which were repeated for each of the 4 users in *U*. In the first two experiments, we used two sets of samples, $S^{+}$ and $S^{-}$, the elements of which differed based on whether they met a certain criterion *C*. We then applied a cross-validation scheme based on the elements in $S^{+}$ over 12,500 iterations. For each iteration, we used 75% of the samples in $S^{+}$ to train an HMM model. The remaining 25% of the samples were used as part of the test set. The distribution of the samples between training and test was randomly calculated for each iteration.

Each time an HMM model θ was built, we computed the probability p(X|θ) for each sample in the test set, which was composed of the remaining 25% of the samples in $S^{+}$ and all the samples in $S^{-}$. The scores produced for each sample in the test set were hence related to the probability that the sample met the specified criterion *C*.

In order to evaluate the effectiveness of the model to discriminate samples according to whether they met the criterion *C*, the results from the 12,500 iterations were used together to build a Receiver Operating Characteristic (ROC) curve and to compute typical accuracy indicators. The accuracy indicators used were the Area Under the ROC Curve (AUC) and the Equal Error Rate (EER). The AUC estimates the probability that the model classifies a random positive sample with a higher rank than a random negative sample. The EER is the point of the ROC curve that corresponds to having an equal probability of misclassifying a positive or negative sample.

In the first experiment, we aimed to test whether the information contained in the physiological signals of an individual can be used to train an HMM model that is able to detect when the subject is concentrated from an unseen sample of the signals. We repeated the experiment for each subject $u_{i}$, always focusing on samples from the same subject. In this case, the criterion *C* was defined as whether the sample was labelled as concentrated. Thus, $S^{+}$ was composed of all concentrated samples for $u_{i}$, and $S^{-}$ contained all non-concentrated samples for the same individual (see the definition of concentrated and non-concentrated samples in Section 3.4).

In the second experiment, we studied whether subjects can be easily distinguished from the others by how concentration affects their physiological signals. This time, we only considered concentrated samples and defined the criterion *C* as whether the sample belongs to a given user $u_{i}$. Thus, again for each $u_{i}$, $S^{+}$ included all concentrated samples for $u_{i}$ and $S^{-}$ included all concentrated samples for the rest of the subjects ($U-\{u_{i}\}$).

Finally, the third experiment attempted to test an inter-subject HMM model: that is, the viability of accurately predicting concentration for an individual $u_{i}$ using data coming from subjects other than $u_{i}$. In this case, we followed a slightly different setting. For each user $u_{i}$, we ran a single validation experiment using all concentrated samples from subject $u_{i}$ as training data. The test set was then built using all concentrated and non-concentrated samples from users in $U-\{u_{i}\}$. The results were assessed using the same measures as in the previous two experiments.

#### 3.5.2. Performance of Physiological Signals on Engagement Assessment

In this second set of experiments, we aimed to evaluate different multimodal fusion at feature level approaches. Thus, within this set of experiments, we wanted to analyse the effectiveness of the use of each physiological signal or a different fusion of them for detection of the concentrated state of the subject.

To this end, we ran the first two experiments of the previous set across each of the 15 possible combinations of the 4 signals in *M*: heart rate, skin conductance, breath rate and skin temperature.

$$\sum_{k=1}^{M}\left(\begin{array}{c} M\\ k \end{array}\right)=2^{M}-1$$

Thus, we analysed the performance of each signal separately as well as the performance of all the possible fusions in an intra-subject approach (one by one M1, in pairs M2 or three by three M3). We repeated each experiment for each subject $u_{i}$. We followed the same definitions as in the previous section to run the experiments regarding the criteria *C* to define samples $S^{+}$ and $S^{-}$, the proportion of the samples across training and test sets, the number of iterations and the way to compute probability p(X|θ).

The purpose of this study was to test whether we can keep detecting the concentrated state of the subject without using all four selected signals. In this case, we wanted to discover which multimodal fusion performs better and which of the rest have a significant performance so it could be considered in future work in order to optimise costs and intrusiveness and thus to maximise the applicability of the detection system. These empirical results will help to make decisions on the selection of sensors to be integrated for the implementation of Commercial-Off-The-Shelf devices in the near future according to accuracy and cost requirements.

## 4. Experimental Results

### 4.1. User-Dependent vs. User-Independent Models

#### 4.1.1. First Experiment

**Figure 2:**
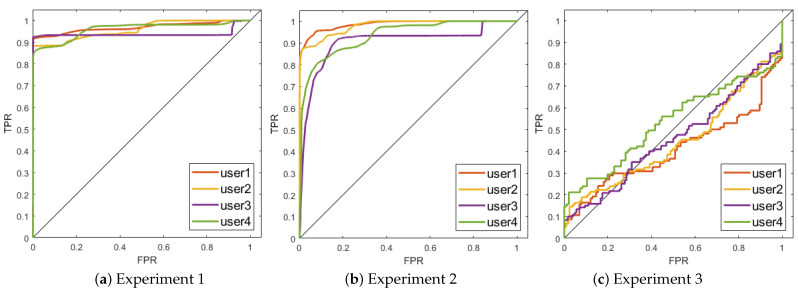
Results for the three experiments carried out represented in Receiver Operating Characteristic (ROC) curves. Experiment 1: using concentrated samples from $u_{i}$ as training data and testing on concentrated and non-concentrated samples from the same user. Experiment 2: using concentrated samples from a user $u_{i}$ and testing on concentrated samples from all subjects in *U*. Experiment 3: using concentrated samples from all users except $u_{i}$ for training, and concentrated and non-concentrated samples from user $u_{i}$ for testing.

Figure 2a represents the ROC curve for the first of the experiments, in which the model was fit by taking 75% of the concentrated samples from one subject and tested against a set that contained both concentrated and non-concentrated samples of the same subject. For each testing sample *X*, the probability p(X|θ) was used as a prediction of whether the user was concentrated. The AUC and EER for each user are shown in Table 3. The lowest AUC is very high, 0.94, and corresponds to the third subject. An average AUC = 0.96 suggests that concentration can be predicted in a very accurate way from the physiological signals when the model has been trained with labelled data from the same subject.

The results are consistent across the 4 users under study and indicate that the HMM model is able to discriminate well between concentrated and non-concentrated samples for the same individual.

#### 4.1.2. Second Experiment

Figure 2b shows the results when the model was trained with 75% of the concentrated samples from a user $u_{i}$ and tested against concentrated samples from all subjects. For each testing sample *X*, the probability p(X|θ) was used as a prediction score of whether the sample belonged to the user $u_{i}$. The high AUC that is shown in Table 3, minimum 0.90, and low error rates obtained in all cases clearly indicate that the way concentration reflects the physiological signals is subject-dependent, to such an extent that, from a concentrated sample, we can accurately find out to which user the sample belongs.

#### 4.1.3. Third Experiment

The relatively higher accuracy values obtained in the second experiment as compared to those obtained in the first one suggests that the subject’s influence in the physiological signals is higher than that caused by the mental state itself. This finding motivated this third experiment to test results when a model is created by using data coming from subjects other than the target. This is, in fact, an inter-subject approach to the detection problem.

Figure 2c shows the ROC curve for each user $u_{i}$ when the model is trained with concentrated samples from all other users and tested against positive and negative samples from user $u_{i}$. The AUC and EER values reported in Table 3 for this experiment are close to a random classifier and reinforce the hypothesis that the subject-related component of the physiological signals is stronger than the concentration-related one and reveal the inadequacy of inter-subject models in this particular context of adaptive systems.

### 4.2. Performance of Physiological Signals on Engagement Assessment

In view of the results obtained in the previous set of experiments and the bad results obtained for inter-subject models (view Section 4.1), the study of the performance for each signal is shown only for the intra-subject models. Thus, in this section, we repeat the first two experiments of Section 4.1 to study the performance of each signal in *M* as well as every combination of them Mk.

#### 4.2.1. First Experiment

**Figure 3:**
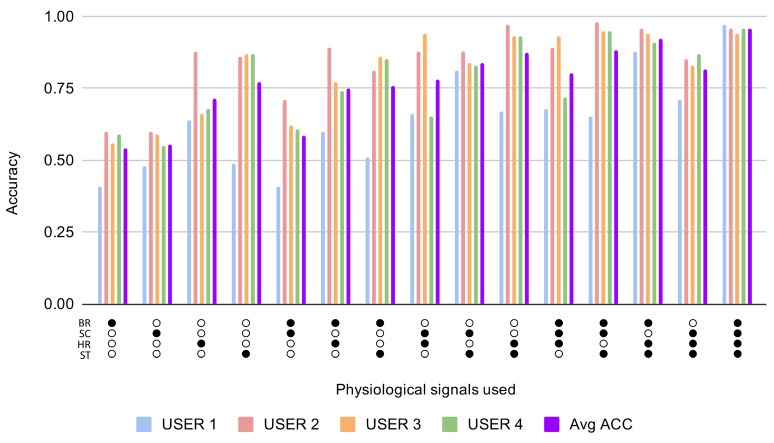
Accuracy results for different combinations of the selected signals for Experiment 1: studying the relevance of physiological features to detect when the subject is concentrated from an unseen sample of such a set of signals: Breath Rate (BR), Skin Conductance (SC), Heart Rate (HR) and Skin Temperature (ST).

Figure 3 shows the results for the first experiment, which test whether the information contained in the physiological signal or signals gathered from each $u_{i}$ can be used to detect when the subject is concentrated from an unseen sample of such set of signals. We repeated the experiment for each subject $u_{i}$, always focusing on samples from the same subject. In the horizontal axis of the figure are represented the set of signals used in each case, and the accuracy obtained for each user as well as the average accuracy from the 4 users are represented in the vertical axis. The numeric results, including AUC and ERR, are detailed in Table 4. The average accuracy results above 0.75, which we consider a relatively high performance that could be enough in some scenarios, have been highlighted with bold text in the table.

**Figure 4:**
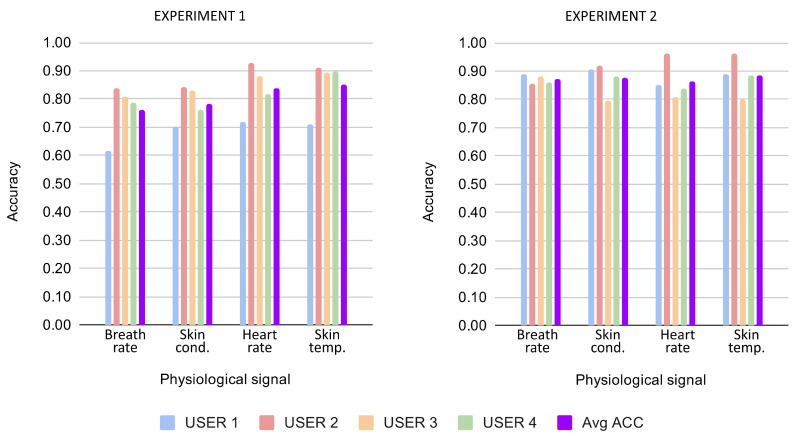
Average accuracy results for both experiments when each physiological signal is present in any of the combinations.

According to the results depicted in Figure 3, when the 4 signals are used separately, skin temperature is usually the signal that provides the most information about the concentrated state of the user. When we fuse any pair of signals, the average accuracy is more than 75% except in the case when we used breath rate and skin conductance. The pair with the highest performance is the one formed by the signals heart rate and skin temperature. When we combined three signals, the behaviour of the model is very similar to that which we obtained with the total set of the four signals. There is only one fusion of three signals that performs a little bit worse than the rest, and that is the one without the skin temperature signal. From the obtained results, we can also deduce that the behaviour of the signals across users become more regular when we combine more signals. As expected, the best result obtained on average is the case using the four signals together. The influence of each signal is also depicted in Figure 4 which shows the average accuracy obtained across all combinations when each signal is present. Results for this first experiment are depicted on the left of the figure, where it is shown how skin temperature obtains again the best performance, really close to the one obtained for heart rate. In summary, from both figures, it can be concluded that a high performance, AUC >0.75, can be obtained to recognise user concentration in the following cases:

- When the skin temperature signal is used independently or present in any of the multi-fusion cases.
- When any of the following pairs of signals are used: heart rate–breath rate, heart rate–skin conductance and heart rate–skin temperature.
- When any possible combination of three of the four selected signals is used.
- When the four signals are used as was already confirmed in previous sections.

#### 4.2.2. Second Experiment

**Figure 5:**
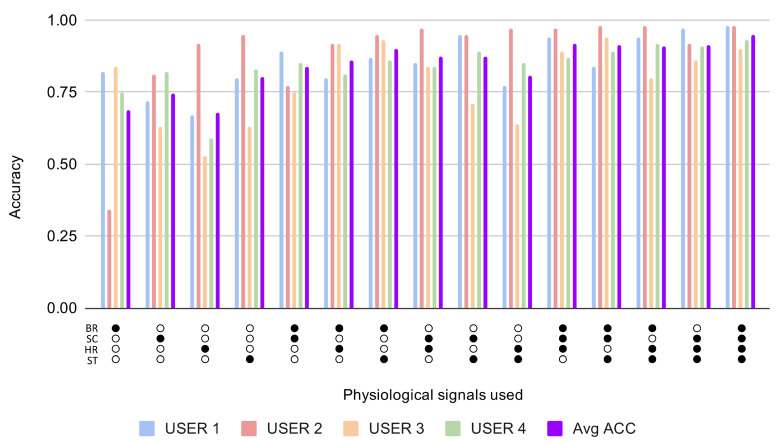
Accuracy results for different combinations of the selected signals for Experiment 2: to study the relevance of the set of signals to discriminate one user from the rest by how concentration affects such a set of signals: Breath Rate (BR), Skin Conductance (SC), Heart Rate (HR) and Skin Temperature (ST).

Figure 5 shows the results for the second experiment, which studies whether subjects can be easily distinguished from the others by how concentration affects different sets of physiological signals. The results are presented in the graph in the same way as the previous experiment. The numeric results of this second experiment are presented in Table 5.

As in the previous experiment, skin temperature showed higher relevance when used separately, obtaining an AUC = 0.80 on average. In this case, all the signals performed better than the previous case when used separately (0.68 for heart rate, 0.69 for breath rate and 0.75 for skin conductance). However, the results across users are less regular, especially when using the BR signal. When using signals in pairs, all the combinations performed an AUC >0.75, with the best pair being the one that includes skin temperature and heart rate, with an AUC = 0.88. When combining the signals in threes, the results are very similar for all combinations, with an average AUC = 0.91 and AUC = 90.92 for the four users. In fact, the performance of the fusion of any three signals is very similar to the one obtained for the set of four signals. The influence of each signal in this experiment is also depicted on the right side of Figure 4 in the same way as previously. In this case, the average accuracy for each signal across all combinations when it is present is very similar. In summary, a high performance, AUC >0.75, can be obtained to discriminate one user from the rest by how concentration affects their physiological patterns:

- When the skin conductance and skin temperature signals are used independently.
- When any possible pair of the selected signals is used.
- When any possible trio of the selected signals is used.
- When the four signals are used as was already confirmed in previous sections.

## 5. Discussion of Results

HMM intra-subject models have been shown to be extremely powerful at detecting concentration. The results reported in the first set of experiments are encouraging and endorse the potential use of HMM models to detect concentration from physiological signals that can be captured by using inexpensive equipment. An average EER of 0.09 when combining the four selected signals and the consistency of the results across all users open the way for further development of low-cost devices that can be integrated into adaptive learning systems, for instance, by using individual models that allow the system to personalise content delivery to increase student’s engagement.

Regarding the relevance of each signal in the detection process, the results reported are also encouraging in some respects. For instance, the use of the skin temperature sensor separately has shown quite a high accuracy in detecting concentration. This sensor is located on the wrist of the subject so that it does not disturb the user while completing any of the tasks. In some scenarios, the accuracy obtained with this sensor could be enough, and although further studies need to be done, the results reported here endorse the potential of this signal to detect concentration in an unobtrusive manner. For scenarios with higher requirements of accuracy, the results showed that any combination of three of the selected signals offers a minimum of AUC = 81%, with a maximum ERR of 0.26. The best result was obtained when using multi-fusion of the four signals, with an AUC of 0.95%. A very similar accuracy, 0.98%, was reported in [55]. However, it is important to note that the scope of this work, the detection of basic emotions, is different from the present one. Instead, the present study aims to detect the concentrated state, which is considered a complex emotion. Furthermore, while other approaches focus on using more signals to maximise accuracy [49,54,59], in this work, we focused on maximising practicality and applicability. Despite the best results being obtained when all 4 signals are simultaneously used, our findings show that user engagement can also be detected with a reasonable accuracy by using a smaller number of sensors. This implies a lower deployment cost, higher user comfort and lower subject reactivity to the measurement context. However, it is also important to notice that the results from the first experiment show that the combined use of more physiological signals makes the results more homogeneous across subjects.

The second experiment indicates that concentration does not manifest in the same way in different individuals. In fact, a subject can be recognised among others by how physiological signals are affected by the concentration state. Two signals, skin temperature and skin conductance, can be used separately to discriminate the concentration state in scenarios that are not very demanding. In this case, the fusion of any pair of selected sensors offers a relatively high accuracy, AUC > 0.84, and a maximum ERR of 0.16. While a lot of previous studies have focused on the recognition of the concentrated state of the students, to the best of our knowledge, none of them deals with recognition of the subject from how concentration affects their physiological signals. Thus, we cannot offer a comparison against previous results by other authors for the second experiment.

Finally, our third experiment outlined the need for intra-subject models to better represent how concentration affects physiological signals and showed that the same model construction process fails when using an inter-subject approach. The findings are directly in line with previous findings in [20]. However, when comparing our results to previous studies that used an inter-subject approach to recognise engaged concentration, as expected, those works with a larger dataset obtained better results. This is the case of [59], where they obtained an accuracy of 63% for a subject-independent model with the fusion of EEG and other peripheral information such as GSR, ST, BR and BVP. It is important to point out that this result was obtained under conditions that were different from the ones in the present work. Regarding the size of the dataset, 20 participants were involved in the experiment. Regarding intrusiveness, the use of EEG signals is far more intrusive and pose strong limitations on the movement of the subjects in real-world scenarios because of the face-mounted electrodes.

Notice that another relevant challenge of intra-subject studies relates to dealing with the relatively small size of the experimental sample. Gathering sufficient data to produce a reasonably accurate model for an individual is a labourious process, which includes several experimental sessions with the same user to capture inter-session variability in the recognised patterns, the participation of more than one expert to ensure consistency in the judgements and interviews with the subject to validate the resulting labels. In the experiments that support the results reported in this paper, we considered all these information sources and made an attempt to maintain data consistency across sessions or activities, but there are still issues that should be analysed in more detail. For example, some of the sessions were held on different days to alleviate the potential effects of fatigue, boredom or tiredness due to long exposure. However, this separation in time may also have a negative effect on signal consistency, as the affective state is also known to have a clear impact on physiological signals [8] and the subject’s mood may significantly vary when sessions are held on different days.

We shall remark that our experiments are very sensitive to potential labelling mistakes, as a few incorrect labels may have a relatively high impact on the result. Despite special care being taken to avoid such mistakes, there is still room for improvement in the labelling methodology by increasing the number of experts and by discarding samples where a full agreement is not achieved. We believe that such an improvement would yield a further reduction in the EER and even better results.

Another issue relates to potential artifacts, which could potentially degrade the quality of the signal or even render it useless. These include not only movement-based one but also others arising from environmental or experimental factors. In the context of this study, environmental events, e.g., external noise, were minimised because the experimental conditions included an isolated setting but noise would appear in a more realistic one. Signal acquisition was implemented using hardware filters as described in [15]. This improvement over other versions of the acquisition system reduces labelling mistakes. With regard to movement-based artifacts, they do not present a major challenge, as concentration is in part characterised by an absence of movement, considered as one of the less “active” states [71], and any symptom of anxiety or uneasiness would dissuade the expert from assigning a concentration label. This argument may also be extended to other different labels when the appearance of a specific move or gesture is associated with its presence or absence. For example, tapping fingers may be related to frustration or to a desire to leave the room. In these sample cases, this kind of artifact actually provides valuable information and it may not be desirable to discard them. Discerning between one type or another of movement and whether they are noise or information is a very interesting issue that would need further and intensive research but would require a different study with a much larger sample size. Despite the interest in this issue, it was left outside the scope of the work presented in this paper, and still results were quite positive for intra-subject models. In spite of that, the most obvious artifacts were filtered in preprocessing of the data, such as negative values of skin conductance due to loss of contact between the sensor and the skin and very high values of heart rate.

Hence, despite the results reported in this paper being reasonably positive, we believe they can be enhanced by applying complementary approaches to increase the quality of the raw data, such as artifact removal methods or specific normalisations that use a daily baseline [20]. This means there is still room for improvement by considering alternative methodologies that help to establish a more robust setting for the purpose of creating intra-subject models.

In addition, rthe esults reported in the paper should be understood as a reference (i) to justify the advantages of using intra-subject models and (ii) to discover the performance of different multi-fusions of several physiological signals towards the development of low-cost and unobtrusive acquisition systems. However, this should not be understood as representative of the performance of this type of models in a practical setting. In such a context, frames should be analysed as part of a sequence rather than as an independent entity, i.e., considering the scores in consecutive signal frames and setting a threshold based on the proportion of positive judgements in the last frames. Such a more informative approach will indeed yield more accurate and consistent results than the ones reported in the experimental section.

## 6. Conclusions and Future Work

One current challenge in user-centred feedback and adaptive systems is the accurate detection of relevant mental states that contribute to improving adaptation capabilities. For practical reasons and in order to achieve solutions that can be widely adopted, it is mandatory to use low-cost and unobtrusive devices and it is also desirable that the required signals can be captured by using wearables.

Some previous works have identified behavioural patterns that are valid across different individuals and can be detected by cameras or eye trackers, e.g., facial muscle activation [72]. However, psychological signals are more affected by subject traits and how emotional, affective, or cognitive states affect their values varying significantly across a population of subjects. In this work, we proved the success of using physiological signals to detect concentration when they are combined with an intra-subject modelling approach and showed the inability of inter-subject models in this particular context. The combination of 4 easy-to-capture signals yielded quite a relatively high performance on detecting the concentration state, and very good results have also been obtained by fusing 3 and even 2 of the selected signals. The benefits of the results presented in this paper extend beyond the AUC and EER values reported, as physiological signals and visual sources provide different types of information and may be used in combination to further improve the already successful rates reported in previous works based on cameras or eye-tracking devices [18]. Together, they allow for practical setups that are minimally intrusive and do not impose any important user mobility limitation.

From a practical point of view, three signals were captured on the subject’s wrists within this work that provided high accuracy in detecting the subject’s concentration. Those signals are heart rate, skin conductance and skin temperature. We can easily envision the possibility of implementing a low-cost wrist-worn device that captures these three signals. The potential advantage of this application is promising to afford better affect-aware experiences in learning scenarios by predicting the student’s engagement. It may be particularly useful in big data studies if devices with an acceptable error rate are achieved by offering a cheap, mobile, nonintrusive and scalable solution as compared with the expensive medical equipment that is commonly used in many works.

The extension to other potential states needs to be explored. For example, a similar approach could be applied to elicit a certain level of frustration and stress by playing with the limitation of time taken for the tasks or their difficulty. Our approach could also be applied to other learning tasks. In fact, the exercises selected during the experiments belong to different learning areas in order to guarantee generalisation of the experiences. Nevertheless, the extension to specific new learning areas would need further study.

Despite the positive results reported in this paper, there are still some ways in which this work can and will be extended. First, there is a need to improve labelling methodologies in order to work with mistake-free data that allows for better estimation of accuracy measures. Second, the available labels in the same data set can be used to build prediction tools for other mental/affective states. Third, despite the negative results using inter-subject models, we consider that they have to be further explored using other alternatives, such as the subject-based normalisation proposed in [20] for EEG signals or mouse and keyboard signals [23]. The development of inter-subject models that can be used on previously unseen subjects is a key issue from a practical perspective and would open the way to seamless integration of this type of technology on today’s learning applications.

In order to further study these aspects and to advance the methodological approach and developments that open the way towards implementing affect-aware user-centred adaptive systems in real-world educational scenarios, we started two new projects funded by the Spanish Ministry of Education. These are ITS-MathPS and INT2AFF. The first of these projects attempts to improve the learning of word problem solving by using a student’s personal cognitive and affective characteristics as a solver, among other data. The second one aims to advance the methodological and practical developments required to address the intertwined relationship between the learner’s affective and cognitive states as the centre and the target of a multisensorial affect-aware user-centred adaptive learning system, which considers the given context in order to provide the most appropriate response to a particular learner in a given situation.

## Figures and Tables

**Table 1:** Summary of the main points of the literature review.

Ref	Emotions	Signals a	Algorithm b	Best. Acc	Dep	N	Labelling
[60]	Engagement/Arousal	GSR	SVM	81%	inter	24	self-report
[48]	Arousal/Valence	EEG, HRV (from ECG)	SVM	75%	inter	60	self-report
[58]	boredom, confusion, curiosity, delight, engagement, frustration, surprise and neutral	ECG, EMG, GSR	SVM, K-nearest, NB, LBNC, LR, C4.5	inter: F1 = 0.41 intra: F1 = 0.62	intra and inter	27	self-report
[59]	boredom, engagement and anxiety	EEG, GSR, BVP, ST, BR	LDA, QDA, SVM	63%	inter	20	self-report
[53]	happiness, surprise, anger, fear, disgust and sadness	EEG	QDA, k-nn, MD, SVM	85.17%	inter	16	self-report
[54]	joy, anger, sadness and pleasure	ECG, EMG, GSR, RSP	C4.5	80%	intra	1	det. by stimulus
[55]	amusement, fear, sadness, joy, anger and disgust	ECG, EMG	MLP, SVM, BN	98%	intra	100	self-report
[56]	amusement, anger, grief and fear	OXY, GSR, HR	Random Forest	74%	inter	101	self-report
[49]	positive/high arousal, negative/high arousal, negative/low arousal and positive/low arousal	EMG, ECG, GSR, BR	LDA	inter: CCR 77 intra: CCR 95	intra and inter	3	det. by stimulus
[50]	5 levels of aurosal and 5 levels of valence	ECG, GSR, BR	QDC	90%	inter	35	det. by stimulus
[61]	3 levels of valence and arousal	ECG, EMG, BR, GSR	KNN, SVM, decision trees	valence: k = 0.35 arousal: k = 0.23	inter	20	self-report

a Signals: (GSR) Galvanic Skin Response; (EEG) Electroencephalogram; (HRV) Heart Rate Variability; (ECG) Electrocardiogram; (EMG) Electromyograph; (BVP) Blood Volume Pulse; (ST) Skin Temperature; (BR) Breath Rate; (OXY) Blood Oxygen Saturation; (HR) Heart Rate. b Algorithms: (SVM) Support Vector Machines; (NB) Naive Bayes; (LBNC) Linear Bayes Normal Classifier; (C4.5) C4.5 Decision Trees; (LR) Multinomial Logistic Regression; (LDA) Linear Discriminant Analysis; (QDA) Quadratic Discriminant Analysis (QDA); (MD) Mahalanobis Distance (MD); (MLP) Multi-Layer Perceptron; (BN) Bayesian Network; (QDC) Quadratic Discriminant Classifier.

**Table 2:** Number of samples per subject.

	Concentrated Samples	Non-Concentrated Samples
subject 1	31	29
subject 2	9	20
subject 3	7	19
subject 4	13	49

**Table 3:** Accuracy results for the first set of experiments (user-dependent vs. user-independent models).

	Experiment 1 (intra)		Experiment 2 (intra)		Experiment 3 (inter)	
**Subject**	**AUC**	**ERR**	**AUC**	**ERR**	**AUC**	**ERR**
1	0.97	0.08	0.98	0.06	0.41	0.54
2	0.96	0.11	0.98	0.09	0.44	0.53
3	0.94	0.07	0.90	0.13	0.45	0.51
4	0.96	0.11	0.93	0.14	0.53	0.43
**Average**	**0.96**	**0.09**	**0.95**	**0.11**	**0.46**	**0.50**

**Table 4:** Results for the first experiment using different sets of signals: Heart Rate (HR), Breath Rate (BR), Skin Conductance (SC) and Skin Temperature (ST).

BR	SC	HR	ST	USER 1		USER 2		USER 3		USER 4		Averages	
				ACC	EER	ACC	EER	ACC	EER	ACC	EER	ACC	EER
●				0.64	0.41	0.88	0.17	0.66	0.36	0.68	0.35	0.72	0.32
	●			0.41	0.59	0.6	0.4	0.56	0.43	0.59	0.43	0.54	0.46
		●		0.48	0.52	0.6	0.45	0.59	0.49	0.55	0.48	0.56	0.49
			●	0.49	0.53	0.86	0.2	0.87	0.16	0.87	0.13	**0.77**	0.26
●	●			0.41	0.56	0.71	0.32	0.62	0.42	0.61	0.38	0.59	0.42
●		●		0.6	0.45	0.89	0.14	0.77	0.21	0.74	0.3	**0.75**	0.28
●			●	0.51	0.51	0.81	0.21	0.86	0.17	0.85	0.21	**0.76**	0.28
	●	●		0.66	0.4	0.88	0.18	0.94	0.08	0.65	0.37	**0.78**	0.26
	●		●	0.81	0.27	0.88	0.15	0.84	0.14	0.83	0.19	**0.84**	0.19
		●	●	0.67	0.34	0.97	0.09	0.93	0.17	0.93	0.13	**0.88**	0.18
●	●	●		0.68	0.41	0.89	0.18	0.93	0.1	0.72	0.32	**0.81**	0.25
●	●		●	0.65	0.4	0.98	0.06	0.95	0.12	0.95	0.11	**0.88**	0.17
●		●	●	0.88	0.2	0.96	0.11	0.94	0.08	0.91	0.16	**0.92**	0.14
	●	●	●	0.71	0.38	0.85	0.21	0.83	0.17	0.87	0.19	**0.82**	0.24
●	●	●	●	0.97	0.08	0.96	0.11	0.94	0.07	0.96	0.11	**0.95**	0.09

**Table 5:** Results for the second experiment using different sets of signals: Heart Rate (HR), Breath Rate (BR), Skin Conductance (SC) and Skin Temperature (ST).

BR	SC	HR	ST	USER 1		USER 2		USER 3		USER 4		Averages	
				ACC	EER	ACC	EER	ACC	EER	ACC	EER	ACC	EER
●				0.82	0.24	0.34	0.65	0.84	0.22	0.75	0.29	0.69	0.35
	●			0.72	0.3	0.81	0.22	0.63	0.45	0.82	0.21	**0.75**	0.30
		●		0.67	0.36	0.92	0.14	0.53	0.51	0.59	0.45	0.68	0.37
			●	0.8	0.29	0.95	0.12	0.63	0.38	0.83	0.2	**0.80**	0.25
●	●			0.89	0.2	0.77	0.29	0.75	0.29	0.85	0.23	**0.84**	0.24
●		●		0.8	0.24	0.92	0.12	0.92	0.16	0.81	0.22	**0.86**	0.19
●			●	0.87	0.22	0.95	0.09	0.93	0.18	0.86	0.2	**0.90**	0.17
	●	●		0.85	0.2	0.97	0.11	0.84	0.21	0.84	0.23	**0.88**	0.19
	●		●	0.95	0.13	0.95	0.13	0.71	0.34	0.89	0.23	**0.88**	0.21
		●	●	0.77	0.29	0.97	0.1	0.64	0.4	0.85	0.2	**0.81**	0.25
●	●	●		0.94	0.11	0.97	0.11	0.89	0.16	0.87	0.16	**0.92**	0.14
●	●		●	0.84	0.24	0.98	0.07	0.94	0.14	0.89	0.15	**0.91**	0.15
●		●	●	0.94	0.1	0.98	0.09	0.8	0.25	0.92	0.19	**0.91**	0.16
	●	●	●	0.97	0.08	0.92	0.11	0.86	0.18	0.91	0.19	**0.92**	0.14
●	●	●	●	0.98	0.06	0.98	0.09	0.90	0.13	0.93	0.14	**0.95**	0.11

## Data Availability

Data sharing not applicable.
